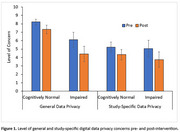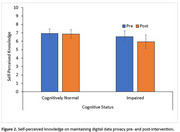# Digital data privacy concerns of cognitively normal and impaired older adults participating in a passive digital monitoring study and response to an educational intervention

**DOI:** 10.1002/alz70856_107144

**Published:** 2026-01-07

**Authors:** Rachel E Mis, Rosemary A Lester‐Smith, Justin Rousseau, June Cui, Ethan Biswas, Yufei Shen, Nayanika Ghosh, Edison Thomaz, Jared F Benge

**Affiliations:** ^1^ The University of Texas at Austin, Austin, TX, USA; ^2^ UT Southwestern Medical Center, Dallas, TX, USA

## Abstract

**Background:**

Passive digital monitoring of real‐world behaviors holds potential for identifying cognitive decline in Alzheimer's disease and related dementias (ADRD). However, little is known about how privacy concerns may affect older adults’ participation in these studies, or how to educate older adults on mitigating privacy risks. This study aimed to characterize digital privacy concerns in older adults enrolling in a passive digital monitoring study and evaluate the efficacy of an educational intervention.

**Method:**

Fifty‐one older adults (mean age: 74.94±5.29 years) enrolled in this study, with 32 classified as cognitively‐normal and 19 cognitively‐impaired (MoCA‐blind cut‐off=18). At baseline, participants answered questions addressing level of general and study‐specific digital data privacy concerns and self‐perceived knowledge on maintaining data privacy. Following a brief educational presentation reviewing data protection measures in this study (e.g., specifying data collected/not collected, instruction on deactivating collection of specific data elements), privacy concerns were re‐assessed. A 2 (Time; pre/post) x 2 (Condition; general/study‐specific) x 2 (Cognitive Status; cognitively‐normal/impaired) mixed model ANOVA analyzed the effect of the educational intervention on privacy concerns, and a 2 (Time) x 2 (Cognitive Status) mixed model ANOVA analyzed its effect on self‐perceived knowledge.

**Result:**

In the privacy concern model, there was a significant within‐subjects effect of Condition, *F*(1,49)=13.69, *p* <.001, η_p_^2^=.22, with greater concern about general versus study‐specific data privacy. A significant Condition x Cognitive Status interaction, *F*(1,49)=4.16, *p* = .05, η_p_^2^=.08, indicated cognitively‐impaired participants endorsed lower concern about general privacy than cognitively‐normal participants. A significant main effect of Time, *F*(1,49)=23.62, *p* <.001, η_p_^2^= .33, indicated privacy concerns were reduced following the intervention. The remaining two‐ and three‐way interactions were not significant, *p*'s >.21. No significant main effects or interactions were found in the knowledge model, *p*'s >.09.

**Conclusion:**

Older adults with and without cognitive impairment expressed greater confidence in their study‐related digital data privacy despite having general privacy concerns. A brief educational intervention effectively reduced participants’ privacy concerns. Results suggest educational efforts by research teams can attenuate digital data privacy concerns in older adults participating in ADRD digital monitoring studies. As digital tools are increasingly incorporated in ADRD studies, it is crucial researchers prioritize participants’ digital data privacy.